# Learning From Others Without Sacrificing Privacy: Simulation Comparing Centralized and Federated Machine Learning on Mobile Health Data

**DOI:** 10.2196/23728

**Published:** 2021-03-30

**Authors:** Jessica Chia Liu, Jack Goetz, Srijan Sen, Ambuj Tewari

**Affiliations:** 1 Department of Statistics University of Michigan Ann Arbor, MI United States; 2 Molecular and Behavioral Neuroscience Institute University of Michigan Ann Arbor, MI United States; 3 Department of Psychiatry University of Michigan Ann Arbor, MI United States

**Keywords:** privacy, data protection, machine learning, mobile health, wearable electronic devices

## Abstract

**Background:**

The use of wearables facilitates data collection at a previously unobtainable scale, enabling the construction of complex predictive models with the potential to improve health. However, the highly personal nature of these data requires strong privacy protection against data breaches and the use of data in a way that users do not intend. One method to protect user privacy while taking advantage of sharing data across users is federated learning, a technique that allows a machine learning model to be trained using data from all users while only storing a user’s data on that user’s device. By keeping data on users’ devices, federated learning protects users’ private data from data leaks and breaches on the researcher’s central server and provides users with more control over how and when their data are used. However, there are few rigorous studies on the effectiveness of federated learning in the mobile health (mHealth) domain.

**Objective:**

We review federated learning and assess whether it can be useful in the mHealth field, especially for addressing common mHealth challenges such as privacy concerns and user heterogeneity. The aims of this study are to describe federated learning in an mHealth context, apply a simulation of federated learning to an mHealth data set, and compare the performance of federated learning with the performance of other predictive models.

**Methods:**

We applied a simulation of federated learning to predict the affective state of 15 subjects using physiological and motion data collected from a chest-worn device for approximately 36 minutes. We compared the results from this federated model with those from a centralized or server model and with the results from training individual models for each subject.

**Results:**

In a 3-class classification problem using physiological and motion data to predict whether the subject was undertaking a neutral, amusing, or stressful task, the federated model achieved 92.8% accuracy on average, the server model achieved 93.2% accuracy on average, and the individual model achieved 90.2% accuracy on average.

**Conclusions:**

Our findings support the potential for using federated learning in mHealth. The results showed that the federated model performed better than a model trained separately on each individual and nearly as well as the server model. As federated learning offers more privacy than a server model, it may be a valuable option for designing sensitive data collection methods.

## Introduction

### Mobile Health Data

The ubiquitous nature of wearables generates considerable potential for data collection and analysis but comes with the issue of protecting user privacy. Some important privacy considerations are protecting patient confidentiality [1], protecting against security breaches [2], and protecting against researchers using user data in a way that the user did not intend [3,4]. Individuals may be more willing to participate in studies and disclose information if these concerns are alleviated [5,6]. Privacy breaches can occur when data servers are compromised but can also occur when data are shared for legitimate purposes by well-intentioned members of the medical community [7]. In this paper, we use privacy to denote the aspects related to protecting the identity, personal information, and use of the data of users.

Mobile health (mHealth) data are often related to the cognitive, behavioral, and affective states of users, making such data highly sensitive. Therefore, we want to ensure that individuals’ confidential health information is not leaked to others. Wearables passively record a range of medically relevant data, such as temperature, heart rate, and electrodermal activity (EDA). As people may carry these items with them throughout the day, this allows for high-frequency collection of data from more people, who may have a greater variety of health conditions, than ever before. Such rich data collection opens up the possibility of using increasingly powerful but data hungry machine learning methods in the analysis of these data [8].

In this paper, we apply predictive machine learning models on the publicly available *Wearable Stress and Affect Detection* (WESAD) data set published by Schmidt et al [9]. In particular, we focus on models that can be trained by fitting a function, potentially nonlinear, to the data using gradient descent and variants thereof. Many of these models make few assumptions about the structure of the underlying data-generating process. To improve privacy, we propose leaving each user’s data on their personal device and training our models using federated learning. In federated learning, there is no single server that contains all users’ information. Instead, model training occurs on each individual’s device, and only model parameter updates leave the user’s device. This allows for more user privacy by maintaining data only on individual user devices. In addition, as explained later, federated learning is still able to take advantage of some shared information across individuals. Thus, it can alleviate some of the concerns in analyzing mHealth data, such as user heterogeneity and privacy preservation.

### Federated Learning

Before further describing federated learning, we establish some common terminology. The goal is to produce a model, often a neural network, trained using data from many individuals. Each individual will have a mobile device, which we call a *user device*—in the networking literature, these are called *clients*—and we will not make a distinction between an individual and their user device. Each user device has its own private data, and the data will never leave the user device. Storing private data on each user device instead of uploading them centrally is the source of improved privacy provided by federated learning. We will also have a single device controlled by the researchers that can communicate with each user device, and we will call the former device the *server.* The server will coordinate the training procedure and store a copy of the model but will not have any private data uploaded onto it. The training process is iterative, as is common for many machine learning models. Each time the model parameters on the server change, we will call that one server training round (or server training iteration).

**Figure 1:**
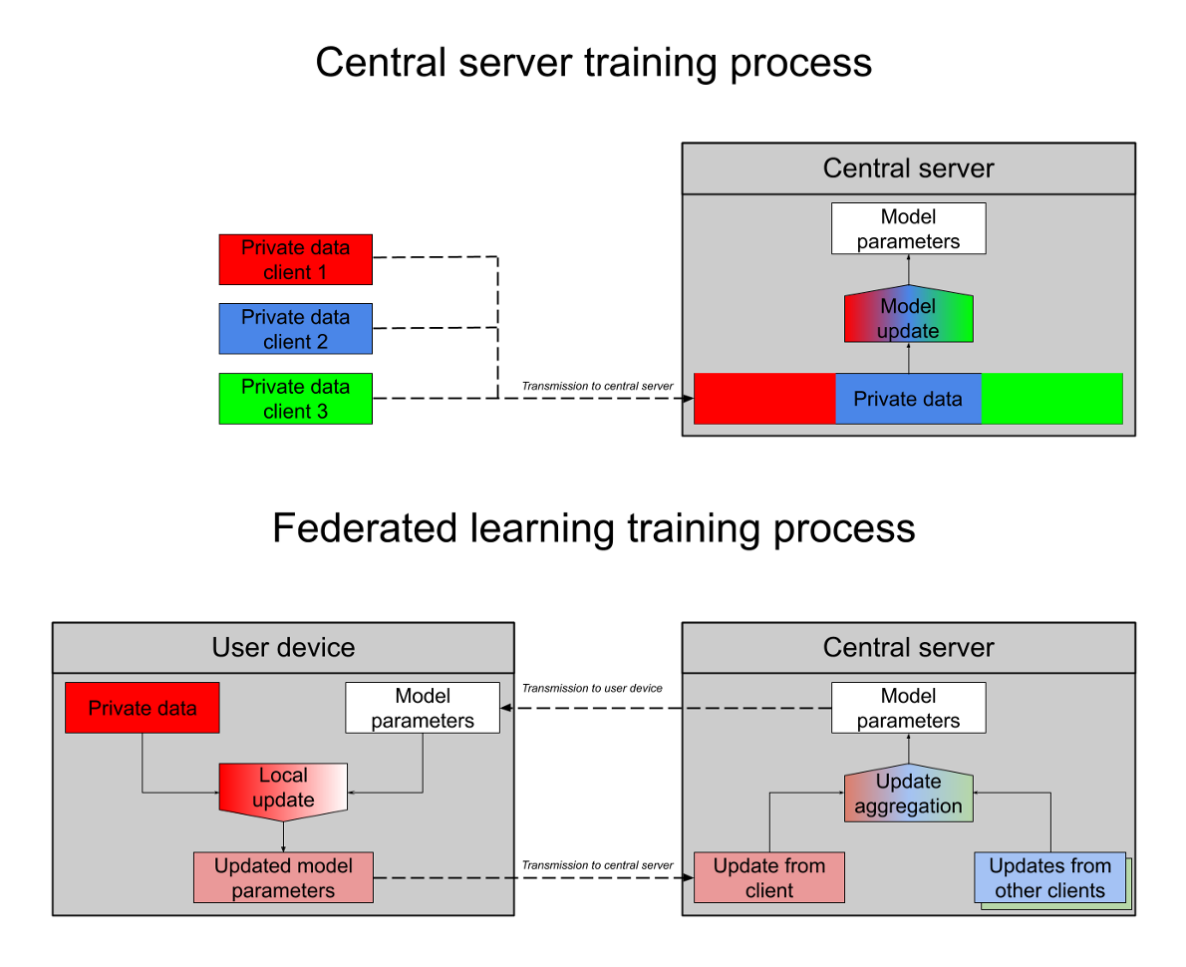
Diagram comparing central and federated learning workflows. Color abstractly represents the private information content at different locations, with red, blue, and green colors representing private information for different user devices. When training on a central server, user data are uploaded onto the server once. In federated learning, model parameters are updated on the user device, producing updates that contain less private information than the data themselves. The updates from many users are then aggregated, further mixing the contribution from each individual user.

In Figure 1, we compare how a model is trained on a central server (top) with how a model is trained using federated learning (bottom). In federated learning, one server training round comprises the following three distinct parts:

1. Broadcast: A small number of user devices will be selected at random, and the current server model will be transmitted to that cohort of user devices.
2. Local update: Each of these user devices will perform a small amount of training of the model they received, using the data from the user device.
3. Update aggregation: Each user device in this cohort will then transmit a copy of their (locally updated) model parameters to the server. The server then averages these parameters and replaces the server model parameters with these new averaged parameters.

**Figure 2:**
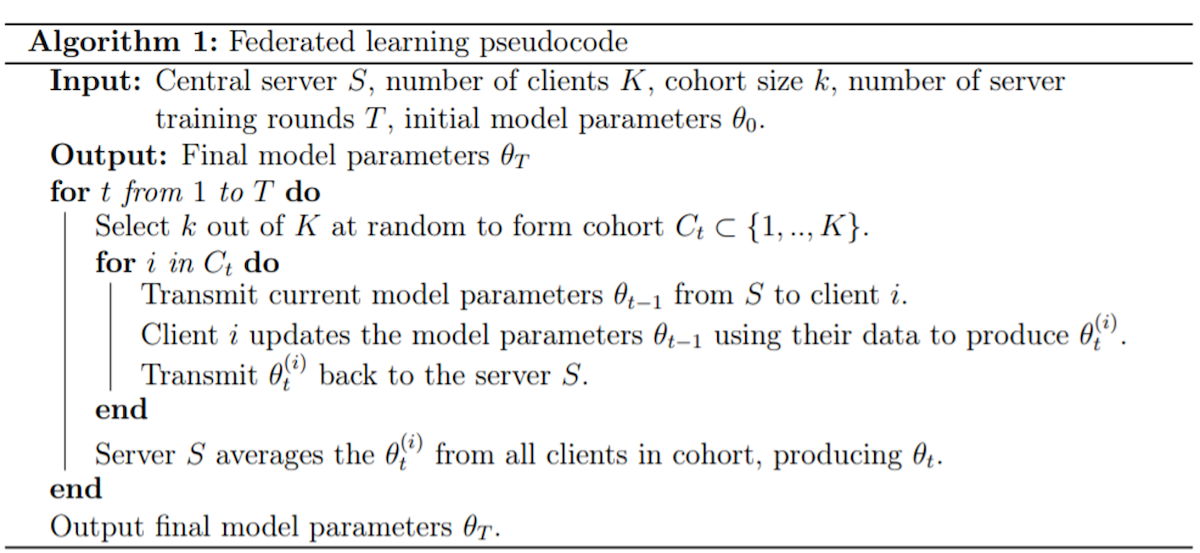
Pseudocode for federated learning algorithm 1.

The server then repeats this process by selecting a new cohort of user devices for each server training round. Thus, the data from every user device contribute to the training of the model. Algorithm 1 (Figure 2) presents the pseudocode, and a full algorithmic description is provided in Multimedia Appendix 1.

The model trains using updates from the data on all user devices; however, individual, private, data remain only with each user device. This protects the privacy of users in several ways. Under federated learning, private data are significantly less vulnerable to the central server being compromised. The server never contains any raw private data and generally contains only the current model parameters. This protects users from being subject to adverse consequences of failings in the server’s security. Another benefit for users under federated learning is private data cannot be reused for other purposes at a later date. It cannot be shared or released either accidentally or in good faith but with inadequate anonymization measures. Finally, federated learning gives users the freedom to withdraw access to their data—both past and future—at any time without relying on guarantees from the researchers that any previously collected data will be deleted.

Federated learning has been successfully applied in complex real-world applications, most notably in training the next-word prediction model for Google’s Keyboard [10]. We refer interested readers to excellent surveys of federated learning [11-13] and federated learning software and data sets [13].

### Previous Work

There has been substantial research on privacy in mHealth and mood prediction using machine learning. Protecting the data of individual users stored in mHealth apps is important because the apps contain personal information about the user, information used to make treatment decisions, and information that could be misused for financial gain, among other concerns [4]. Users’ willingness to disclose personal information in mobile data varies according to their demographics and personal characteristics [14-16]. Concerns about privacy vary among different age groups; for example, some older people are less willing to use mHealth services [6,14] and are more likely to cite privacy concerns [6]. As such, it is important to consider the target population when considering the amount of privacy guarantee needed. Increased privacy concerns about health information technologies reduce patients’ willingness to share information and reduce their positive attitudes toward the technologies [14,17]. In the same vein, reducing privacy concerns makes patients more comfortable in sharing their health information [5,17]. Sometimes, individuals are willing to give up some privacy if they consider mHealth technology as beneficial; however, this depends on the sensitivity of the information they are providing [18]. Due to these privacy concerns, there are many innovations for increasing patient trust and privacy protection [19].

Many researchers have used machine learning methods, including neural networks, random forests, and support vector machines, to predict outcomes such as depression, mood, and stress using mobile data [20-26]. Various physiological and behavioral features help predict these outcomes, such as skin temperature, EDA, 3-axis accelerometer data, mobility, sleep, and self-reported histories [20-23,27,28]. Detecting aberrations in some of these measures can help identify early signs of mental illness [16].

Personalization techniques in machine learning allow parts of a model to be specific to each user while other parts are shared between users, allowing both the sharing of data to estimate certain parameters and fitting to unmeasured covariates of each individual. To an extent, both unique traits among different individuals and commonalities across users are helpful in prediction. Some studies have taken advantage of this and grouped users based on common traits, finding that this improved mood prediction accuracy [8,24,29]. In the same vein, accounting for individual user differences is valuable in prediction using machine learning [8]. There is growing recognition within the mHealth literature [30] that there are trade-offs between individual and centrally trained models and that often there are reasons to make them work in tandem.

In the Methods section, we describe federated learning in the context of mHealth by addressing some of the issues raised in the mHealth literature. Specifically, we discuss the application of federated learning using neural networks to make predictions from mHealth data. We address practical considerations for researchers who consider using federated learning for their study. We then evaluate the performance of federated learning on an mHealth data set to predict subjects’ affective states.

## Methods

### Federated Learning in mHealth

Federated learning is a nascent field within machine learning [31], which arose, at least partially, in response to the opportunities and difficulties presented by personal devices that can record information and perform computation [11]. As such, many practical challenges in mHealth, such as privacy [32,33], user heterogeneity [34], user hardware constraints [35,36], and even incentivizing voluntary participation [37], are already being studied by the machine learning community. The medical science community has also proposed ways to share information while preserving privacy, such as developing models in a distributed manner [38,39], using a federated patient hashing framework for similar patient matching [40], and using collaborative privacy-preserving training to detect protected health information in text [41]. A recent paper describes how federated learning can be useful to various stakeholders in the mHealth field [42].

Federated learning provides a middle ground between extremes in privacy and data utilization. One extreme is collecting data centrally on the server and performing all analyses and training there. The other extreme is individual training, in which each user device trains a completely separate model on its own data.

In centralized learning, we take all individual users’ raw data and store them in one location. This unlimited access gives researchers the most flexibility when analyzing the data—clearly any analysis that can be performed with limited data access can be performed with unlimited data access—and the best chance of extracting useful insights from the data. However, it is likely that some individuals are reluctant to have all of their raw data stored in a central location, as the central location could be hacked or because individuals feel that their data are too sensitive to be released. A model trained using federated learning is useful because it is trained without putting all individuals’ raw data together in one place. Not only does this help alleviate concerns about data privacy, but it can also help potential subjects be more willing to engage in studies, as their raw data will not be sent to a foreign central location. As we show in our case study, a model trained in a federated way performs almost as well as a centrally trained model. We refer to the former as the federated model and the latter as the server model.

In individual training, each user device trains a separate model using only their own data; therefore, no information, besides potentially their final model, leaves the user device. We refer to this as the individual model. This provides a very high level of privacy. However, it prevents information sharing across users during model training. Federated learning takes advantage of the information shared across users, which can be especially useful when analyzing health care data. Each person’s characteristics and health care need personalization; however, there is also a lot of useful information that can be shared across people, as every person’s distribution is not entirely different. In training, the algorithm can learn from similar users and apply this knowledge when predicting another user’s outcome. It is significantly easier to use a federated model to make predictions for a new patient because there is a single model that is trained using data from many people, and we do not need previous data from the new patient. In this way, the federated model can do better than the individual model. Thus, federated learning can help improve the prediction accuracy while preserving privacy.

### Practical Considerations for Using Federated Learning

The decision to use federated learning must be made when designing the study so that one leaves the data on the user devices and trains the model using federated learning. If data have already been collected from each user device and stored together on a server, privacy has been violated, and we cannot use federated learning to retroactively make the experiment privacy preserving. A small burden might need to be placed on user devices to use federated learning. To minimize disruptive usage of user device bandwidth and compute resources, the local update on the user device should take place while the user device is connected to a nonmetered internet such as Wi-Fi, and the device should be idle and plugged into a charger. To avoid inducing bias in the training process by the overuse or underuse of certain user devices because of their passive adherence to these restrictions, we may need to request that user devices be available for local training during certain times of the day. The structuring of these times should be considered during the experimental design phase. In addition, the server should follow certain protocols to minimize the privacy loss. The model updates may contain some information about the data on the user device; however, the privacy loss from these updates can be mitigated by not storing or viewing any locally updated parameters during each server training round. Similarly, information about when each user device participated in the training should also not be stored.

Although concerns about user privacy deter users from sharing health information, the perceived effectiveness of information security reduces these concerns [5,17]. However, federated learning is not the only tool for privacy, and it does not alone provide perfect privacy. Depending on the privacy requirements of the study, federated learning may or may not be the best method to use. We provide a few examples to illustrate cases where federated learning may not be the correct method.

- Low privacy: If a doctor is collecting data from his or her patients and the patients completely trust their doctor and the security of their server, then there is no need to collect data using federated learning—the doctor can simply take all of his or her patients’ raw data. However, the more assured patients are about their information security, the more willing they are to disclose information and the higher their perceived quality of care [17].
- Verifiable privacy: If patients completely distrust the researcher, they may insist on formal guarantees of privacy. This could manifest itself as requiring privacy protection from an honest-but-curious server, wherein the server will attempt to learn anything it can from the information it receives. Under such a requirement, we cannot simply transmit model parameters back to the server, as an individual’s model parameters may reveal information about the user. It requires significant technical expertise and engineering effort to implement federated learning under such a strong privacy requirement [43], which may be beyond the resources available in a clinical trial.
- Privacy under model access: Even if no user device data are transmitted to the server, it may be possible to infer information about the data used to train the model, given sufficient access to the model itself, including the reconstruction of specific data points used to train the model [44]. Protection against such attacks is particularly important if the model will be made available to those beyond trusted researchers; in the most extreme cases, researchers themselves may not be trusted. Federated learning provides no guarantees of protection against such attacks; complementary forms of privacy protection, such as differential privacy [45], may be required.

Federated learning is not a silver bullet against privacy loss, and it is not a black-box tool that can be simply tacked onto an existing study. However, when properly integrated into the study design, federated learning can significantly reduce the loss in users’ privacy while incurring only a small reduction in model accuracy.

### Evaluation of Federated Learning on mHealth Data

To assess the practicality of using federated learning on mHealth data, we compared its predictive performance with that of other predictive machine learning models. We used the WESAD data set for this purpose [9]. We had a 3-class classification problem using physiological and motion data to predict whether each subject was, at the time, performing a task designed to elicit a neutral, stressed, or amused affective state.

We used the WESAD data set because it is publicly available and thus easily accessible by other interested researchers, and it is in the University of California, Irvine, Machine Learning Repository as a data set that measures stress in users using wearables. The data were collected from 15 subjects and contained physiological and motion data measured simultaneously by a wrist device and a chest device during specific tasks designed to capture 3 different affective states: neutral, stress, and amusement. There was approximately 36 minutes of data for each subject. Using 30-second windows for all 15 subjects, 1087 windows were generated in total. Of these windows, 53.45% (581/1087) were collected during the baseline task, 29.99% (326/1087) during the task designed to elicit stress, and 16.56% (180/1087) during the task designed to elicit amusement.

The authors of the data set found that using physiological and motion data from the chest-worn device was more informative than using physiological and motion data from the wrist-worn device in the 3-class classification problem [9]. The chest-worn and wrist-worn devices differed slightly in the modalities they measured. Thus, we restricted our analysis to the data collected using the chest-worn device. These include electrocardiogram, EDA, electromyogram, respiration, body temperature, and 3-axis acceleration (x-axis, y-axis, and z-axis) measurements collected at 700 Hz. For additional information, we refer interested readers to the original WESAD paper [9].

The authors of the WESAD paper used feature extraction to identify useful features for their predictive models, which often included the mean, SD, minimum, and maximum of measurements. For simplicity, we used these four summary statistics, calculated over 30-second windows, of each of the 8 measurements for each subject as the features in our models. The code to extract our features was derived from Matthew Johnson’s GitHub repository [46]. The limitations of our analysis include whether the subject was in the intended affective state and the set of features used.

As we had access to each subject’s data, we were able to use a server model. However, in many situations, researchers may not want to access each subject’s raw data for privacy reasons. As true federated learning would not store raw data on a central server, we could not use a true federated model and instead use simulated federated learning. Nonetheless, we saw this as a feature and not a limitation. As we had the server data, we could compare the accuracy between our simulated federated model and the truth in the centralized data. If we had implemented true federated learning, we would not be able to compare performance with the centralized, or server, model, or compare performance with the individual model. Much of published federated learning research uses federated learning simulations for experimental results [31,32,34,36].

#### Data Preprocessing

Of the 15 participants, 12 were male and the remaining 3 were female. Table 1 shows the demographics of the subjects in the WESAD study. Table 2 shows the summary statistics of some of the features used. The full table of summary statistics for the features used is provided in Multimedia Appendix 1.

**Table 1:** Demographic characteristics of the participants in the study (N=15).

Characteristic	Value, mean (SD)
Age (years)	27.5 (2.4)
Height (cm)	177.6 (6.7)
Weight (kg)	73.1 (10.3)

**Table 2:** Summary statistics of a subset of features.

Feature	Participants, first quartile	Participants, median	Participants, mean (SD)	Participants, third quartile
Mean EDA^a^	2.0	3.7	4.6 (3.4)	6.3
Mean ECG^b^	7.2E−04	1.1E−03	1.1E−03 (7.8E−04)	1.5E−03
Mean EMG^c^	−3.5E−03	−3.0E−03	−3.0E−03 (9.2E−04)	−2.5E−03
Mean respiration	−0.02	0.05	5.4E−02 (2.0E−01)	0.13
Mean temperature	34	34	34 (1.3)	35
Mean ACC_X^d^	0.73	0.86	8.0E−01 (1.3E−01)	0.90
Mean ACC_Y^e^	−0.06	−0.02	−3.1E−02 (1.0E−01)	0.02
Mean ACC_Z^f^	−0.54	−0.31	−3.5E−01 (2.6E−01)	−0.17

^a^EDA: electrodermal activity.

^b^ECG: electrocardiogram.

^c^EMG: electromyogram.

^d^ACC_X: 3-axis acceleration (x-axis).

^e^ACC_Y: 3-axis acceleration (y-axis).

^f^ACC_Z: 3-axis acceleration (z-axis).

#### Neural Network Architecture

We tuned the hyperparameters for the individual, federated, and server models separately. We used 3-fold cross-validation to jointly tune the number of epochs and learning rate for the individual and server models. For the federated model, because it involves more hyperparameters, we jointly tuned the number of epochs, learning rate, number of clients sampled per round, number of local updates per round, and step size. The training set consisted of the first two-thirds of the data from each task for each user. The test set consisted of the remaining third of the data from each task for each user. Within each training set, one-third of the training data were used for validation, that is, to tune the hyperparameters.

Our neural networks had 1 dense input layer with 12 hidden units, followed by a dense layer with 10 hidden units, and then followed by a dense layer with 8 hidden units. These layers used the leaky rectified linear unit (leaky ReLU) activation function with a slope of 0.01 where the x-axis is negative. The output layer was a dense layer with 3 hidden nodes and used a softmax activation with categorical cross-entropy loss. We used Adam as the optimizer. We standardized our training data and applied that same standardization to our test set. By privately calculating the mean and SD, standardization can preserve privacy [47]. Our code, which uses Python TensorFlow, and the best hyperparameters chosen by our cross-validation process are available on the authors’ GitHub repository [48]. The transformed data are available on GitHub and in Multimedia Appendix 2 [9].

**Figure 3:**
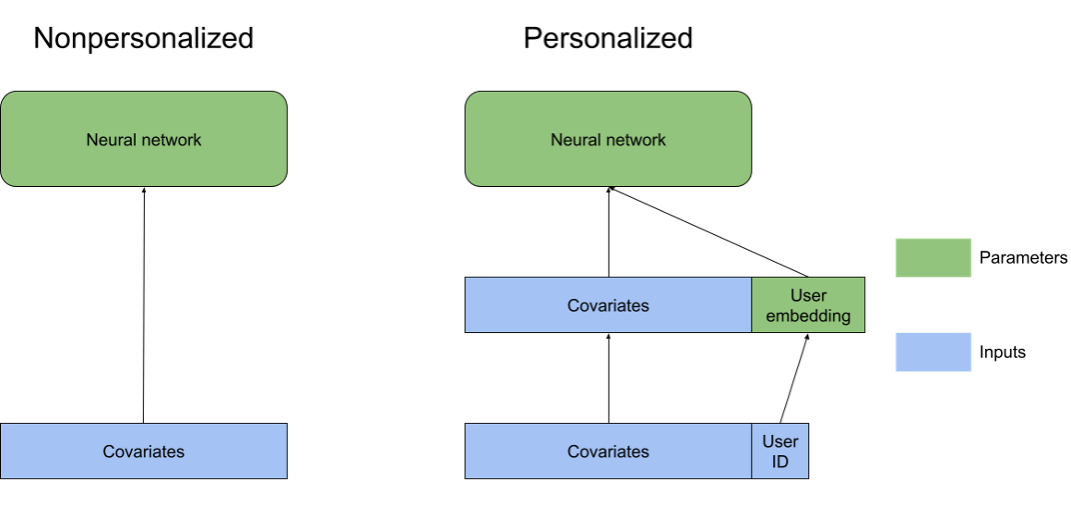
Comparison between the architecture of nonpersonalized and personalized versions of our models. The green blocks represent trained model parameters, and the blue blocks represent the input covariates. Note the user ID is removed from the covariates once it is used to attach the correct parameters, which represent that user’s embedding.

We tested augmentations in the architecture to allow for model personalization. The simplest example of adding personalization to a model is a fixed effects model for linear regression, where each user is given a user-specific intercept, and the other parameters are estimated jointly. This is mathematically equivalent to augmenting each data point by adding a single covariate, where the covariate’s value is the same for all data points from the user, and the value is learned by the model-fitting process. We add personalization to our neural network in a similar way, in which we augment each data point by adding a user embedding*,* with *u* extra covariates that are the same for all data from that user and learned by the model-fitting process (Figure 3). Note that if we solely use user embeddings, then each prediction for the same user would be exactly the same, and the best possible prediction would be the user mean for all data points. This is a very simple example of collaborative personalization [49,50]; in the same way fixed effects models can be extended, there are many extensions of collaborative personalization. However, this simple method is sufficient for our study.

User embeddings often encode unmeasured user-specific covariates, meaning that access to user embeddings from a trained model has the potential to expose information about that user. However, a small change in the federated learning protocol can prevent such privacy loss. As user embeddings are user-specific and only updated by training with the user’s data, there is no need to transmit gradients for updating any user embeddings to the server. Thus, each person’s user embedding may be kept only on that user’s device, preventing the server from using that embedding to learn private information about that user.

To choose the dimension of the user embedding for the personalized federated and personalized server models, we used 3-fold cross-validation, as described earlier, with 1D, 2D, and 3D user embeddings. We used the same number of dimensions for both personalized models each time. Using a 2D user embedding achieved the highest accuracy on the validation set for the personalized server model. As such, we used 2D user embeddings in our final personalized federated and personalized server models.

## Results

In this section, we present the results for evaluating federated learning on the WESAD data set and compare them with the performance of a server model and an individual model. We include results of personalized federated and server models as well as nonpersonalized federated and server models.

There is inherent variation each time we run a neural network because the initial weights are randomized, and there is randomness in stochastic gradient descent as the neural network tries to find the local minimum. Further, there is variation each time we run a federated model because federated learning takes a random sample of user devices per round of training. As such, we ran each of our models 15 times, using different seed settings each time. We report the median and mean accuracy over 15 tests in Table 3. The distribution of the results over 15 tests and graphs of the average prediction accuracy after each epoch of training of the neural network are presented in Multimedia Appendix 1.

**Table 3:** Median and mean accuracy of each model over 15 tests.

Model	Accuracy, median	Accuracy, mean (SD)
Personalized server	0.929	0.932 (0.019)
Server	0.897	0.888 (0.028)
Personalized federated	0.929	0.928 (0.018)
Federated	0.853	0.859 (0.021)
Individual	0.899	0.902 (0.021)

The personalized server model achieved the highest accuracy. The personalized federated model came in second, performing nearly as well as the personalized server model. Thus, the personalized server model and the personalized federated model beat their nonpersonalized counterparts.

The individual model outperformed both the nonpersonalized server and nonpersonalized federated models. The individual model is trained separately on each user, whereas the server model takes the data of all individuals at once and cannot adjust for each user. For comparison, a model that always predicts the majority class would attain 53% accuracy on the data set.

These results provide evidence that using personalization, which takes into account individual differences, helped improve the prediction accuracy of the models. This is reasonable for the WESAD data set, considering that each person has varying baseline physiological measurements.

## Discussion

### Principal Findings

This paper discusses the advantages and challenges of using federated learning as a predictive model in mHealth data collection and demonstrates an empirical example in which federated learning could have been effective. Furthermore, it shows empirical evidence that a federated model can make accurate predictions, is compatible with personalization, and has the added privacy advantage of not storing raw data from individual users. The personalized server model performed the best on the chosen data set, followed closely by the personalized federated model. As a federated model offers more privacy for users than a server model, there is evidence to suggest that federated learning may be a valuable option for collecting and analyzing sensitive mHealth data.

### Limitations

The decision of whether to use federated learning is based on a combination of factors, including how much privacy is required, how much data are available, and what resources are available to implement the federated model. Each of the models we tested has advantages and disadvantages. The server model uses all raw data stored in one place for easy access and future use. However, this poses the risk of breaching user privacy if the server is compromised and the risk of the data being accessed and used in a way that the users did not intend. An individual model can maintain user privacy by keeping the data on the user’s device and requires less engineering to implement than a federated model. However, the individual model did not perform as well as the personalized server model and personalized federated model in our tests. Nonetheless, if a researcher has a lot of data from each user, using an individual model can be useful for prediction, and a federated model may be unnecessary. The federated model provides more privacy than a server model, as well as reasonably accurate predictions, but it requires some design work before collecting data and software engineering to implement. Moreover, although federated learning provides an added degree of privacy, it does not guarantee privacy, as explained in the Methods section. To achieve stronger privacy guarantees in federated learning, a substantial amount of software engineering is needed.

Federated learning is used for predictive modeling; therefore, implementing it limits the research questions that can be answered from the data. Researchers may often wish to pursue research questions aside from prediction; in the future, it would be interesting to extend federated learning to estimate treatment effects. Other future work includes developing ways to handle missing data for time series in the context of federated learning. In addition, Python TensorFlow has recently released capabilities to apply federated learning in their TensorFlow Federated package, and it would be interesting to compare it with our implementation.

### Comparison With Previous Work

Previous research in the mHealth field has explored privacy-preserving methods. Some of these studies do not involve neural networks and federated learning [38-40]. One study applied a methodology similar to federated learning [41], although it selectively updated parameters from individual users, whereas we used multiple local updates between transmission rounds. As federated learning started in the machine learning community, there have been papers in the computer science and engineering fields regarding collaborative privacy-preserving learning tested on mHealth data [33,51]. Our work bridges the mHealth and machine learning communities by discussing practical considerations for mHealth researchers who want to consider implementing federated learning. Furthermore, we evaluate the effectiveness of federated learning on a publicly available mHealth data set. Our code is available on GitHub to encourage reproducibility of our results.

As mentioned earlier, many mHealth researchers have applied machine learning methods to predict mood and stress [20-23,25,26]. Research has shown that accounting for both unique traits among different individuals and commonalities across users is valuable in prediction [8,24,29]. We demonstrate the use of federated learning as a privacy-preserving method that also has these advantages.

### Conclusions

This paper discusses federated learning and its importance in the field of mHealth. For example, federated learning provides added protection to preserve patient confidentiality and inhibits the use of data in a way that the participants in a study did not intend. As federated learning does not store raw data from individual users on a central server, there is no possibility of a central server being hacked and raw data leaked. This provides more privacy to users when recording sensitive data.

Protecting user privacy is critical in mHealth. Having more privacy protection measures in place may encourage people to participate in a study and to be more willing to disclose information that is useful for treatment and research. Federated learning offers additional data privacy and can overcome some common challenges in mHealth data by addressing user heterogeneity and taking advantage of commonalities across users. As such, federated learning has considerable potential to help advance mHealth research.

## Appendix

Multimedia Appendix 1 Federated learning algorithm, summary statistics, and additional figures.

Multimedia Appendix 2 Transformed data.

## Abbreviations

EDA: electrodermal activity
mHealth: mobile health
WESAD: Wearable Stress and Affect Detection
