# Supplementary material for: Learning From Others Without Sacrificing Privacy: Simulation Comparing Centralized and Federated Machine Learning on Mobile Health Data
Source: JMIR Mhealth Uhealth. 2021 Mar 30;9(3):e23728. doi: 10.2196/23728 (PMC8044739; doi:10.2196/23728)
Supplement: Multimedia Appendix 1 [file mhealth_v9i3e23728_app1.pdf]

Appendix

Federated learning full algorithm

Federated learning restricts the allowed data interaction during training, meaning certain models may be difficult or even impossible to train. Fortunately any models which can be trained using gradient descent (which includes models from linear and logistic regression to neural networks) can be trained with federated learning with minimal adjustment to the local training process on each user device (client).

Algorithm 2: Federated Learning

Input: Central server S, number of clients K, cohort size k, number of server training rounds T, initial model parameters θ_0 with $|\theta_0| = m$, client data sets $\{\mathcal{D}_i\}_{i=1}^{K}$, function $localUpdate : \{\mathcal{X} \times \mathcal{Y}\}^{\mathbb{Z}} \times \mathbb{R}^m \rightarrow \mathbb{R}^m$, function $aggregateUpdates : \{\mathbb{R}^m\}^k \rightarrow \mathbb{R}^m$.

Output: Final model parameters θ_T

for t *from* 1 *to* T **do**
    Select cohort $C_t \subset \{1, .., K\}, |C_t| = k$ uniformly at random.
    for i *in* C_t **do**
        Transmit current model parameters θ_{t-1} from S to client i.
        Client i locally computes $localUpdate(\theta_{t-1}, \mathcal{D}_i) = \theta_t^{(i)}$.
        Transmit $\theta_t^{(i)}$ back to the server S.
    end
    Server S computes $aggregateUpdates(\{\theta_t^{(i)}\}_{i \in C_t}) = \theta_t$.
end
Output θ_T.

The most simple function for *localUpdate* is to run a single round of gradient descent and return the updated parameters. In practice people usually do multiple epochs of SGD. The most simple function for *aggregateUpdates* is to take the mean, and in practice this works well.

Summary Statistics of Features

The features we used are the mean, standard deviation (SD), minimum, maximum of each physiological and accelerometer measurement. Each feature is calculated over 30-second windows for each subject.

**Measurement**	**Mean**	**SD**	**First Quartile**	**Median**	**Third Quartile**
EDA_mean	4.59	3.45	1.98	3.72	6.31
EDA_SD	0.04	0.07	0.01	0.02	0.03

EDA_min	4.41	3.43	1.80	3.54	6.18
EDA_max	4.76	3.50	2.10	3.85	6.49
ECG_mean	1.10E-03	7.82E-04	7.24E-04	1.14E-03	1.54E-03
ECG_SD	0.26	0.06	0.23	0.27	0.30
ECG_min	-0.60	0.23	-0.75	-0.54	-0.42
ECG_max	1.42	0.20	1.49	1.50	1.50
EMG_mean	-2.96E-03	9.21E-04	-3.51E-03	-2.97E-03	-2.54E-03
EMG_SD	1.30E-02	5.91E-03	9.55E-03	1.14E-02	1.49E-02
EMG_min	-0.10	0.08	-0.11	-0.08	-0.06
EMG_max	0.09	0.08	0.04	0.06	0.11
RESP_mean	0.05	0.20	-0.02	0.05	0.13
RESP_SD	3.24	1.61	2.11	2.84	3.90
RESP_min	-7.36	4.66	-8.96	-5.92	-4.16
RESP_max	8.68	5.55	4.77	7.09	10.73
TEMP_mean	33.76	1.32	33.54	34.12	34.53
TEMP_SD	0.05	0.58	0.02	0.03	0.03
TEMP_min	33.35	9.40	33.44	34.01	34.41
TEMP_max	33.94	1.30	33.72	34.28	34.69
ACC_x_mean	0.80	0.13	0.73	0.86	0.90
ACC_x_SD	1.37E-02	1.96E-02	5.61E-03	8.84E-03	1.58E-02
ACC_x_min	0.73	0.26	0.67	0.78	0.84
ACC_x_max	0.88	0.16	0.80	0.91	0.97
ACC_y_mean	-0.03	0.10	-0.06	-0.02	0.02
ACC_y_SD	1.90E-02	2.46E-02	5.93E-03	1.09E-02	2.23E-02
ACC_y_min	-0.11	0.23	-0.15	-0.09	-0.04
ACC_y_max	0.04	0.13	-0.01	0.05	0.10
ACC_z_mean	-0.35	0.26	-0.54	-0.31	-0.17
ACC_z_SD	3.13E-02	3.95E-02	1.12E-02	1.91E-02	3.52E-02
ACC_z_min	-0.45	0.31	-0.64	-0.44	-0.28
ACC_z_max	-0.21	0.32	-0.45	-0.19	3.20E-03

Additional Figures

Pers. refers to personalized

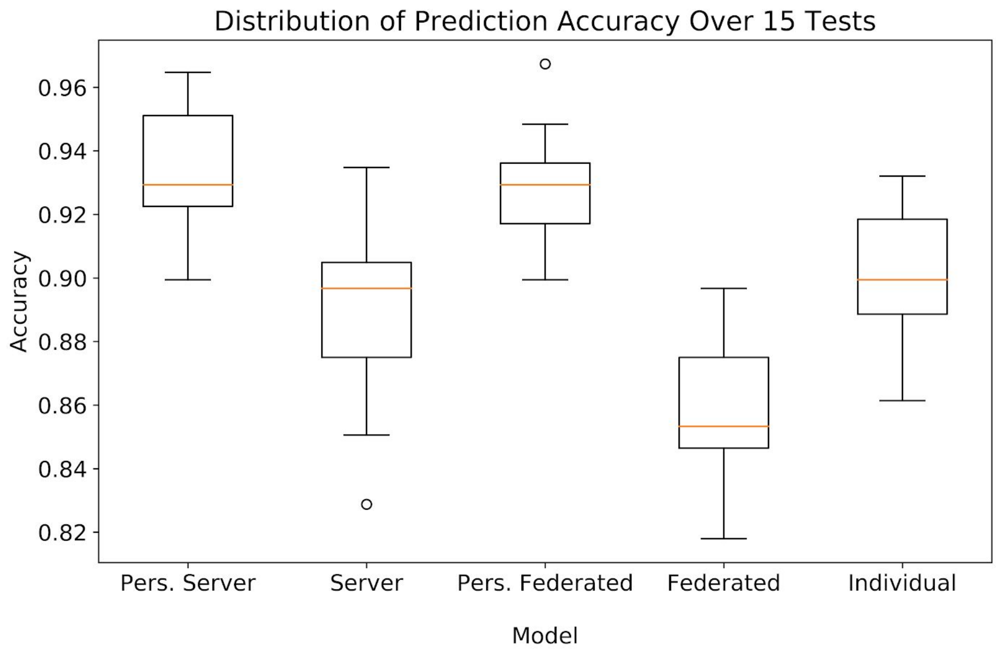

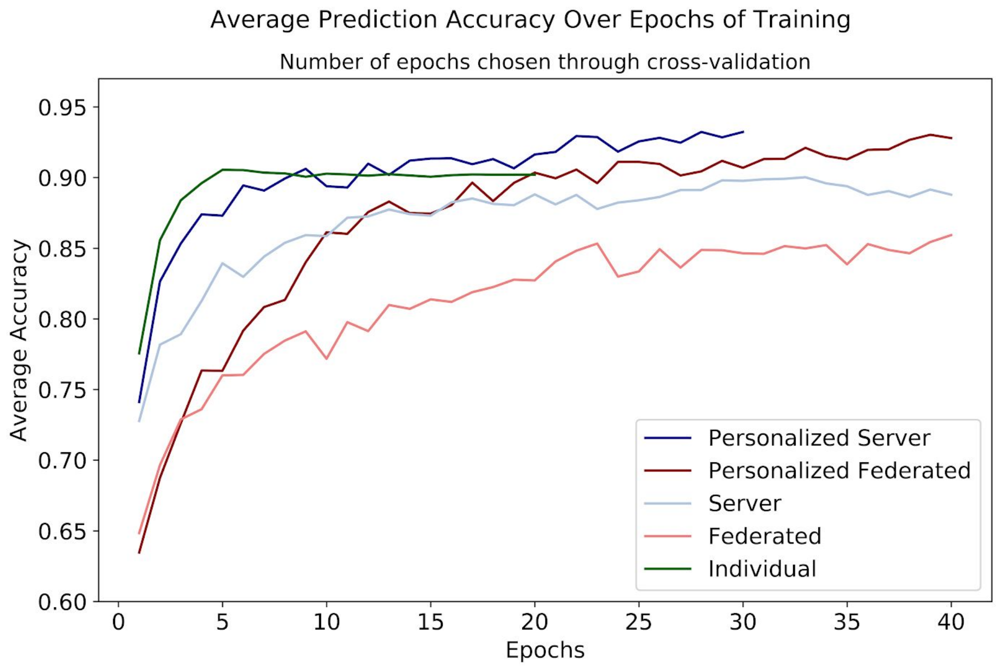

Average Prediction Accuracy By User

User refers to the subject's ID. Two IDs were removed by the authors of the WESAD dataset due to a malfunction in the data collection.

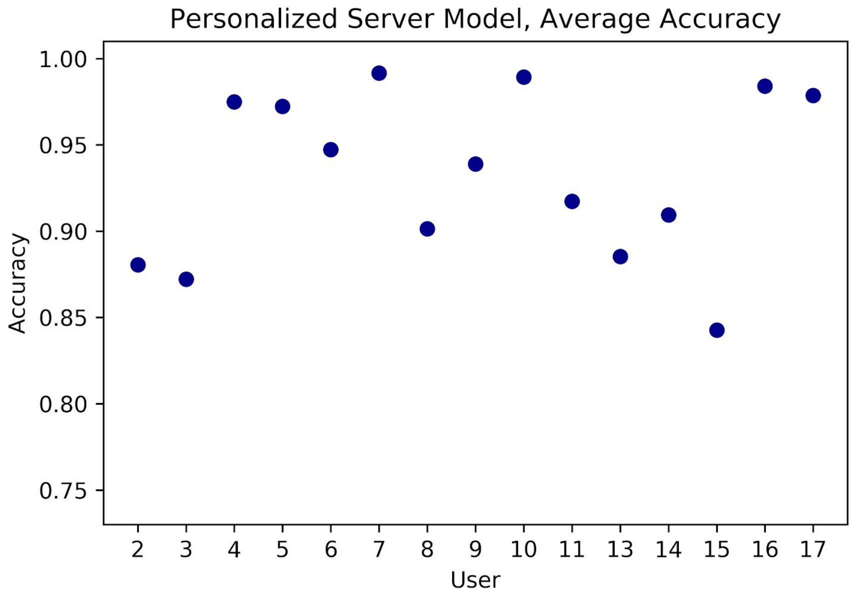

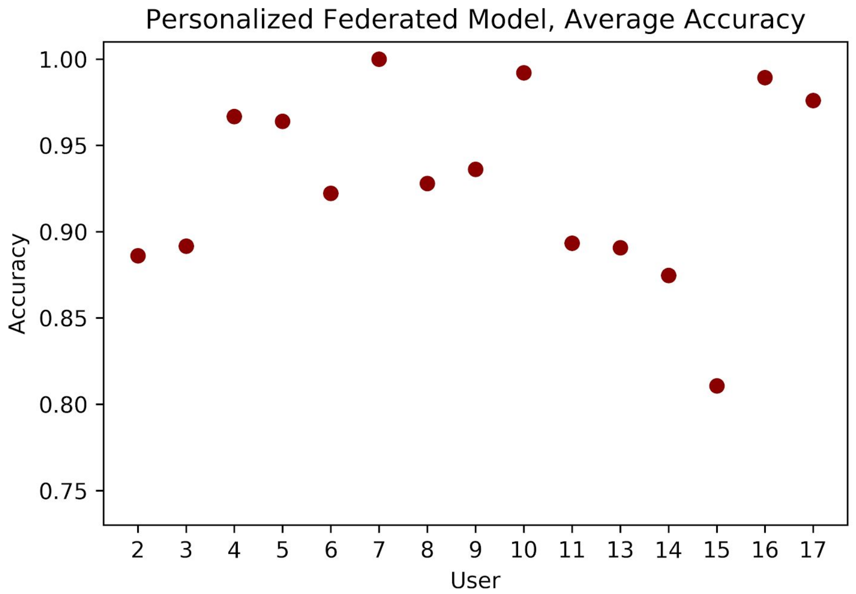

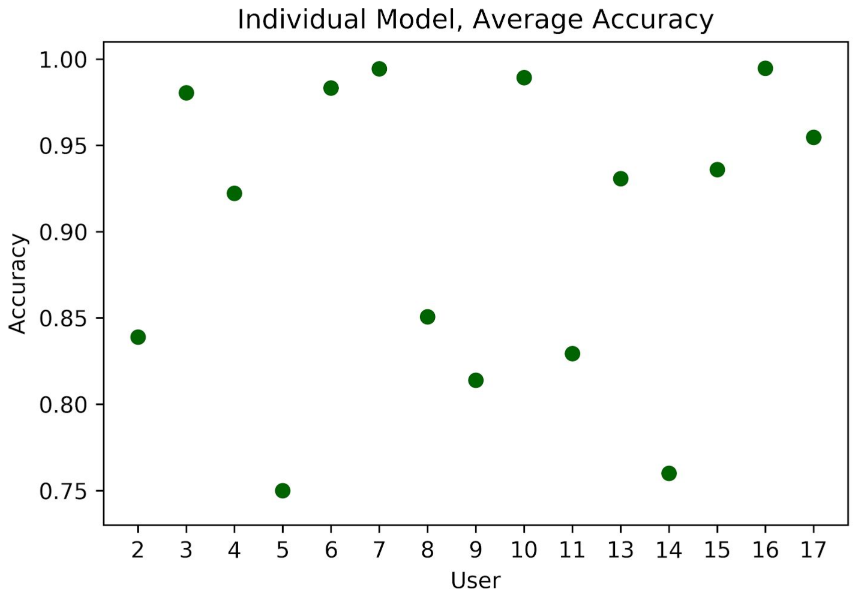
Individual Model, Average Accuracy
Accuracy
1.00
0.95
0.90
0.85
0.80
0.75
2 3 4 5 6 7 8 9 10 11 13 14 15 16 17
User

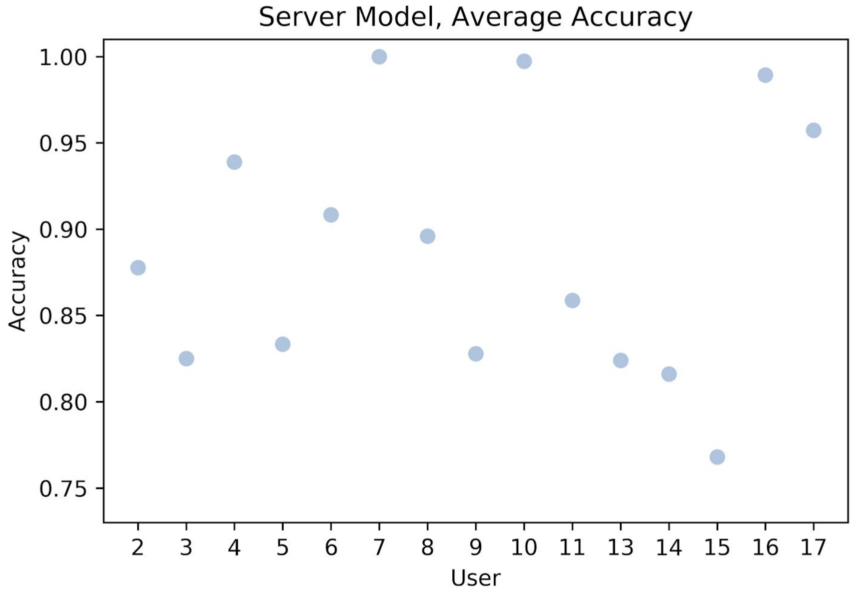
Server Model, Average Accuracy
Accuracy
1.00
0.95
0.90
0.85
0.80
0.75
2 3 4 5 6 7 8 9 10 11 13 14 15 16 17
User

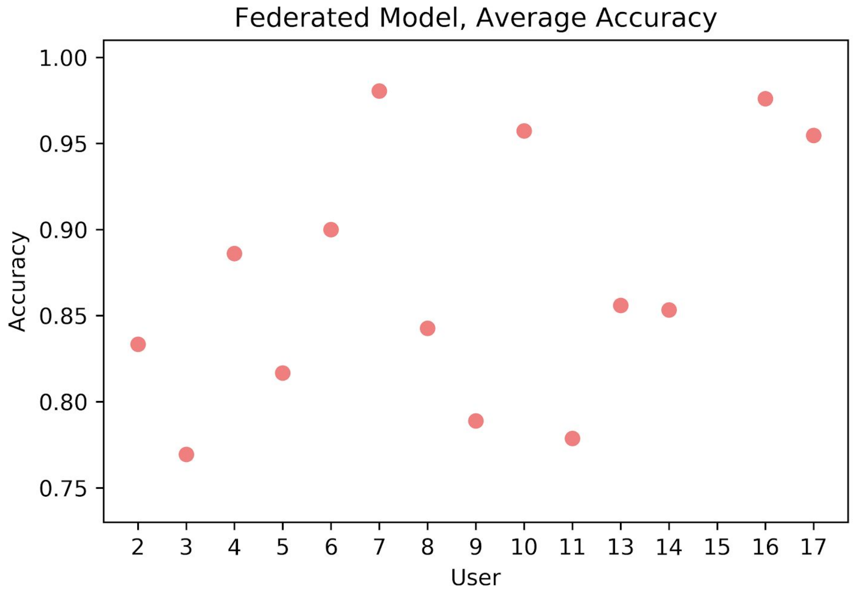
Federated Model, Average Accuracy
Accuracy
1.00
0.95
0.90
0.85
0.80
0.75
2
3
4
5
6
7
8
9
10
11
13
14
15
16
17
User
